# Diversity of cultivated aerobic poly-hydrolytic bacteria in saline alkaline soils

**DOI:** 10.7717/peerj.3796

**Published:** 2017-09-25

**Authors:** Dimitry Y. Sorokin, Tatiana V. Kolganova, Tatiana V. Khijniak, Brian E. Jones, Ilya V. Kublanov

**Affiliations:** 1 Winogradsky Institute of Microbiology, Research Centre of Biotechnology, Russian Academy of Sciences, Moscow, Russia; 2 Department of Biotechnology, Delft University of Technology, Delft, Netherlands; 3 Institute of Bioengineering, Research Centre of Biotechnology, Russian Academy of Sciences, Moscow, Russia; 4 DuPont Industrial Biosciences/Genencor International BV, Leiden, Netherlands; 5 Immanuel Kant Baltic Federal University, Kaliningrad, Russia

**Keywords:** Aerobic, Soda solonchak soils, Hydrolytics, Haloalkaliphilic, *Bacillus*, *Actinobacteria*

## Abstract

Alkaline saline soils, known also as “soda solonchaks”, represent a natural soda habitat which differs from soda lake sediments by higher aeration and lower humidity. The microbiology of soda soils, in contrast to the more intensively studied soda lakes, remains poorly explored. In this work we investigate the diversity of culturable aerobic haloalkalitolerant bacteria with various hydrolytic activities from soda soils at different locations in Central Asia, Africa, and North America. In total, 179 pure cultures were obtained by using media with various polymers at pH 10 and 0.6 M total Na^+^. According to the 16S rRNA gene sequence analysis, most of the isolates belonged to *Firmicutes* and *Actinobacteria*. Most isolates possessed multiple hydrolytic activities, including endoglucanase, xylanase, amylase and protease. The pH profiling of selected representatives of actinobacteria and endospore-forming bacteria showed, that the former were facultative alkaliphiles, while the latter were mostly obligate alkaliphiles. The hydrolases of selected representatives from both groups were active at a broad pH range from six to 11. Overall, this work demonstrates the presence of a rich hydrolytic bacterial community in soda soils which might be explored further for production of haloalkalistable hydrolases.

## Introduction

Alkaliphilic aerobic hydrolytic bacteria have already attracted attention for a long time as sources of alkali-stable hydrolases for various industrial applications, primarily enzymatic laundry detergents (reviewed by: Horikoshi, 2004; Horikoshi, 2006; Fujinami & Fujisawa, 2010; Grant & Heaphy, 2010; Sarethy et al., 2011; Zhao, Yan & Chen, 2014; Mamo & Mattiasson, 2016). Most of this research has been conducted with non-halotolerant *Bacillus* species producing alkalistable proteases, amylases and endoglucanases. In contrast, only a few salt tolerant alkaliphilic hydrolytics have been isolated and characterized from saline alkaline (soda) lakes. So far, the majority of known soda lake hydrolytics belonged to fermentative anaerobic bacteria. A low salt-tolerant *Clostridium alkalicellulosi* is so far the only truly anaerobic cellulolytic bacterium able to grow on crystalline cellulose found in soda lakes (Zhilina et al., 2005). Pectin utilization for growth at haloalkaline conditions has been demonstrated in two fermentative anaerobic haloalkaliphiles: *Natronoflexus pectinovorans* (*Bacteriodetes*) and *Natranaerovirga hydrolytica* (*Clostridia*) at moderate and high salinity, respectively (Sorokin et al., 2011; Sorokin et al., 2012a). Two groups of fermentative haloalkaliphilic bacteria, narrowly specialized in the utilization of chitin as a growth substrate, have been found in hypersaline soda lakes. They formed two classes, *Chitinivibrionia* (high salt-tolerant) and *Chitinispirilla* (low salt-tolerant) within the phylum *Fibrobacteres* (Sorokin et al., 2012b; Sorokin et al., 2014; Sorokin et al., 2016). *Proteinivorax tanatarense* (*Clostridiales*), isolated from the soda lake decaying phototrophic biomass, represents a so far single example of anaerobic proteolytic haloalkaliphilic microorganism (Kevbrin et al., 2013).

Very few examples of aerobic hydrolytic haloalkaliphiles have been characterized from soda lakes, with most of the work done on alkaline protease producers. The low to moderately salt-tolerant organisms are represented by a well-studied salt-tolerant gammaproteobacterium *Alkalimonas amylolytica*, producing amylase (Ma et al., 2004), *Alkalibacillus* sp. (*Firmicutes*), *Nesterenkonia* sp. (*Actinobacteria*) and *Salinivibrio* sp. (*Gammaproteobacteria*) producing haloalkalitolerant serine proteases (Abdel-Hamed et al., 2016; Gessesse et al., 2003; Lama et al., 2005), as well as several *Gammaproteobacteria* from the genus *Marinimicrobium* and a number of *Actinobacteria* strains, utilizing chitin (Sorokin et al., 2012b). Furthermore, a unique group of aerobic extremely halo(alkali)philic hydrolytic *Euryarchaeota* is also present in hypersaline soda lakes. The previous findings characterized highly haloalkalistable protease-producing *Natronococcus occultus*, *Natrialba magadii, Natronolimnobius innermongolicus* (Studdert et al., 2001; de Castro et al., 2008; Selim et al., 2014) and amylolytic *Natronococcus amylolyticus* (Kobayashi et al., 1992). Recently we also demonstrated a presence of four novel genus-level groups of natronoarchaea in soda lakes capable of growth on insoluble celluloses and chitin (Sorokin et al., 2015).

However, another type of mainly aerobic soda habitats, saline alkaline soils, also called soda solonchaks, remain practically unexplored as a potential source of aerobic haloalkaliphilic hydrolytics. In contrast to the mostly anoxic soda lake sediments, soda soils are well aerated and remain desiccated most of the year. Such conditions should favour predominance of aerobic spore-forming *Firmucutes* and *Actinobacteria*, as has been shown in our recent exploration of bacterial nitrogen fixation in such habitats (Sorokin et al., 2008). Soda solonchaks are located in patches in dry steppe and semi-desert areas, such as south Siberia, north-eastern Mongolia, northern China, Egypt, India, Pakistan, Hungary and North American Steppes. In many cases they are hydromorphic and associated with high-standing saline, alkaline ground waters and often occur in the vicinities of saline alkaline (soda) lakes (Bazilevich, 1970; Kondorskaya, 1965).

In this paper we describe a previously unexplored culturable diversity of aerobic haloalkalitolerant hydrolytic bacteria recovered from saline alkaline soils of several regions in Central Asia, Africa and North America.

## Materials and Methods

### Sample characteristics

Surface soil samples (0–5 cm depth) were collected into sterile plastic Petri dishes at five locations in Central Asia, Egypt and California. Each individual sample comprised a composite of 4 subsamples randomly collected in a 3–5 m^2^ area. Samples from Kenya and Tanzania were collected in sterile plastic bags (Whirl-Pak®; Nasco, Fort Atkinson, WI, USA) and vials using disposable sterile tongue depressors as described previously (Duckworth et al., 1996). The samples were kept at 4 °C before analysis. At most locations, the top soil layer was desiccated at the sampling time with a 20% maximum content of moisture. The selection of the samples was based on an immediate measurement of pH of a 1:5 water extract using a field pH-conductivity meter (model WTW 340i; WTW, Weilheim, Germany). Only those soils showing the pH of the water extract above 9.5 were selected for sampling. In total, more than 70 saline alkaline soil samples were obtained. Some of their characteristics are presented in Table 1. The content of total soluble salts was estimated in the laboratory by gravimetry after extraction of 2 g dry soil homogenized with 5 ml water followed by filtration through 0.2 µm filter and drying at 105 °C. Carbonate alkalinity in the soluble fraction was determined by acid titration monitored by a pH meter, using 5 g dry soil extracted with 20 ml water and after centrifugation at 10,000 × g for 10 min a 10 ml aliquot was titrated to pH 4.5 with 0.1 M HCl providing the value of total soluble carbonate alkalinity (NaHCO_3_ + Na_2_CO_3_).

**Table 1:** Characteristics of soda solonchak soils and lacustrine dry soda mud samples.

General information				pH of 1:5 water extract	Total soluble salts (g/kg)	Soluble carbonate alkalinity (mM)
Sample code	Number of samples	Year of sampling	Sample type			
AA	10	1988	SS	9.45–10.2	12–388	20–1,870
KUS	4	1998	SS	9.2–9.9	26–96	23–40
BS	2	1998	SS	9.71–10.70	25–60	10–502
KS	14	2003	SS	9.60–10.21	53–385	150–1,520
MS	24	1999	SS	9.70–10.80	12–128	10–1,140
EWN	3	2000	SS	10.05–10.30	85–102	750–1,740
MLC	4	2001	SLM	9.2–9.8	30–43	130–240
KT	16	1988; 1996; 1999	SLM	9.6–10.7	43–160	45–890

**Notes.**

Sample code AA Ararate valley Armenia BS Barabinskaya Steppe, Novosibirsk region, Russia KUS Kunkurskay steppe, Buriatia, Russia KS Kulunda Steppe, Altai region, Russia MS north-eastern Mongolia, Choibalsan province EWN Wadi al Natrun valley, Libyan Desert, Egypt MLC Mono Lake, California, USA KT Kenya-Tanzania Sample type: SS continental soda solonchak soil SLM dry soda mad near soda lakes

### Enrichment, isolation and cultivation of pure cultures of haloalkaliphilic aerobic hydrolytic bacteria

The general methods for the cultivation of aerobic alkaliphiles have been described elsewhere (Grant, 2006). The basic sodium carbonate mineral medium for cultivation of moderately salt-tolerant alkaliphiles contained 0.6 M total Na^+^ and 1 g l^−1^ K_2_HPO_4_ and was strongly buffered at pH 10. After sterilization, the medium was supplemented with 1 mM MgSO_4_7H_2_O and trace metal solution (Pfennig & Lippert, 1966). The enrichments were performed in 20 ml medium contained in 100 ml bottles closed with rubber septa (to prevent evaporation during prolonged incubation) inoculated with 1 g soil. Incubation was performed on a rotary shaker at 100 rpm and 28 °C. After achieving growth and polymer degradation, the cultures were plated on solid media of the same composition. Five different polymers were used as substrates at concentration 1 g l^−1^: CMC, soluble starch, casein, powdered alpha-keratin and emulsified olive oil prepared according to Sorokin & Jones (2009). Testing of pure cultures also included 3 additional polymers: beech xylan, amorphous cellulose and chitin prepared as described by Sorokin et al. (2015). In the case of CMC, xylan and olive oil, the solid medium was supplemented with 0.2 g l^−1^ and in the case of chitin and starch—with 20 mg l^−1^ yeast extract. Growth of the xylanase-positive cultures on xylan was also tested in liquid culture containing 20 mg l^−1^ yeast extract. The pure cultures were isolated from individual colonies and checked for purity by repeated re-inoculation on to solid media. The culture purity and endospore formation was also checked by phase contrast microscopy (Zeiss Axioplan Imaging 2; Zeiss, Göttingen, Germany) and, finally, by nucleotide sequencing. The pH profiling of growth and hydrolytic activities was performed on solid media containing 0.6 M total Na^+^ in the form of either NaCl (for pH 5–8) or NaHCO_3_–Na_2_CO_3_ (for the pH range 8–11). The media at pH range 5–8 were buffered with a mixture of potassium phosphates (50 mM) and HEPES (50 mM).

### Detection of hydrolytic activities

All activities were detected using plate assays. Beta-1,4-endoglucanase and endoxylanase activities were visualized by using sequential flooding of the plates with 0.1% (w/v) Congo Red and 1 M NaCl each with 30 min incubation (Teather & Wood, 1982). The hydrolysis of keratin, emulsified olive oil, and amorphous chitin and cellulose was directly observed by formation of clarification halos around the colonies (Sorokin & Jones, 2009; Sorokin et al., 2015). The hydrolysis of starch was visualized after flooding the plates with 0.05 N J_2_ solution, containing 1% KJ. The hydrolysis of casein was visualized by flooding the plates with 10% (w/v) trichloroacetic acid. For several strains the pH profile and thermotolerance of endoglucanase activity were measured in culture supernatant by agar diffusion approach and measurements of reducing sugar release with DNS (Miller, 1959).

### 16S rRNA gene sequence and analysis

Genomic DNA was extracted from colony biomass using alkaline SDS cell lysis at 65 °C for 30 min followed by pH neutralization and DNA purification using the Wizard MaxiPreps Purification resin (Promega, Madison, WI, USA). For this, the following steps were taken: (1) cell material taken from solid medium was resuspended in 100 µl of buffer I; (2) 125 µl of lyzing buffer II was added and the resulted mixture was vortexed and (3) incubated at 65 °C for 30 min; (4) 125 µl neutralizing buffer III was added, the resulted mixture was vortexed, centrifugated at 10,000 g for 10 min; (5) 200 μl of the Wizard MaxiPreps resin (Promega) was added to the supernatant and next purification steps were made according to the Wizard DNA Extraction System manufacturer‘s instructions. The final DNA concentration was generally > 10 m kg ml^−1^, D_260_:D_280_ > 1.8, RNA contamination was less than 1%. Buffer I: 50 mM Tris–HCl, pH 8.0, 10 m M EDTA, 50 µg/ml pancreatic RNAse. Lyzing buffer II: 1% SDS in 0.2 M NaOH. Neutralizing buffer III: 2.5 M CH_3_COOK, pH 4.5. The 16S rRNA gene was amplified with bacterial forward primer 11f and the reverse universal primer 1495r. Sequencing was performed commercially using standard Sanger sequencing techniques. The obtained sequences were analyzed using SILVAngs web interface (Quast et al., 2013) on 07.03.2017. The Project summary and settings are shown in Table S1. The 16S rRNA gene sequences of 13 isolates, possibly representing novel taxa, together with the most identical sequences from the Ganbank, verified by BLASTn, were aligned in MAFFT 7 (Katoh et al., 2002). The Maximum Likelihood phylogenetic analysis with General Time Reversible model (*G* + *I*, 4 categories, Nei & Kumar, 2000) was performed in MEGA 6 (Tamura et al., 2013).

## Results

### Isolation and identification of pure cultures of aerobic hydrolytics from saline alkaline soils

**Figure 1:**
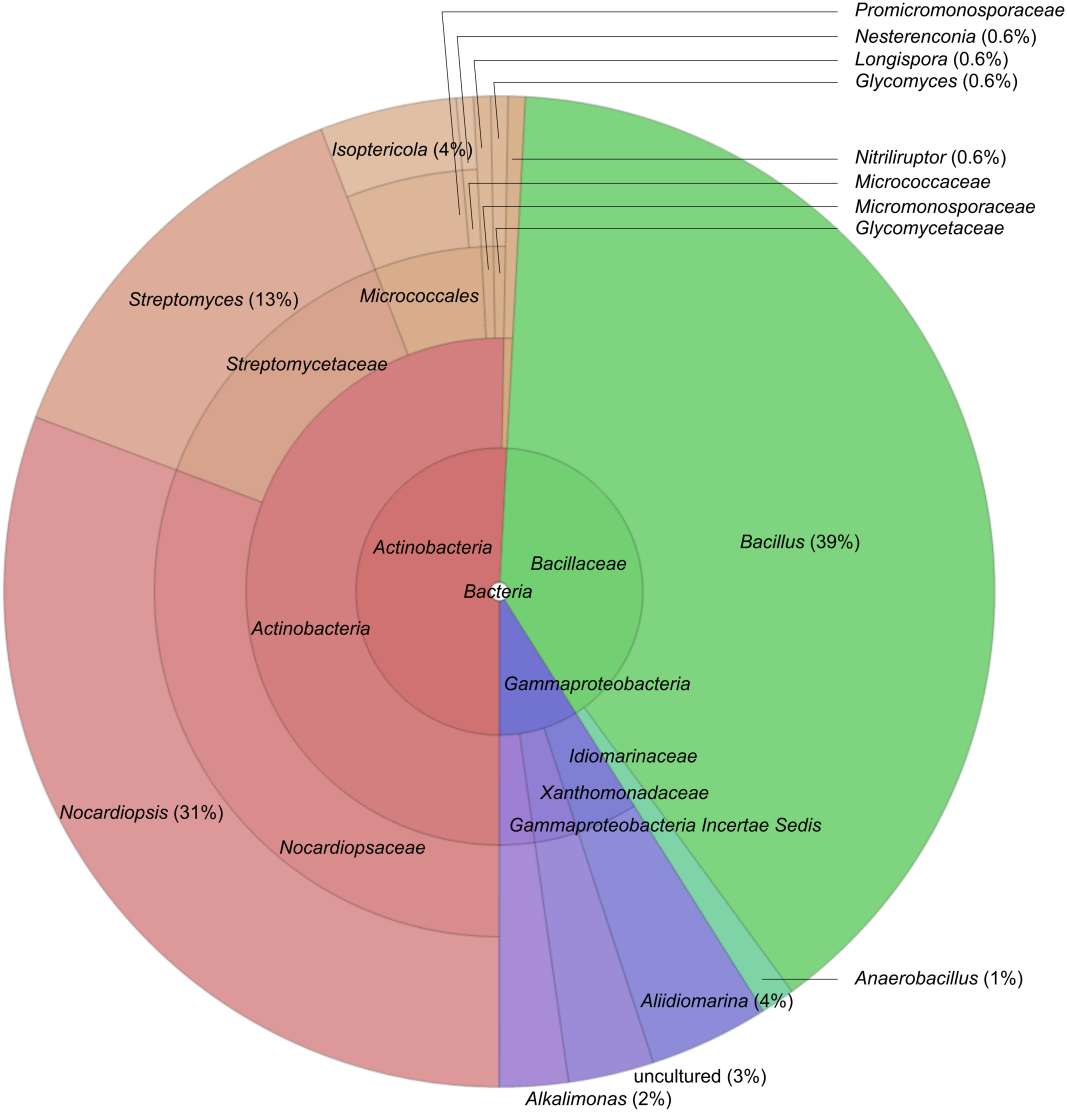
Distribution of 179 almost complete 16S rRNA gene sequences of hydrolytic haloalkaliphilic bacterial isolates, created by SILVAngs service.

A total of 179 strains with one of five polymer degrading activities have been isolated. From the general colony morphology and microscopy, the isolates were obviously dominated by two large groups—actinomycetes (formation of aerial or substrate mycelium) and endospore-forming bacilli. Furthermore, isolates obtained with proteins as substrate also included Gram-negative bacteria. The identification by 16S rRNA gene sequencing generally confirmed this conclusion. The two largest groups of isolates from the saline soda soils are typical hydrolytics belonging to the phyla *Actinobacteria* and *Firmicutes* (Fig. 1, Table 2) which may reflect a combination of the specific habitat (Table S2), sampling methods and culture conditions (Duckworth et al., 1996).

**Table 2:** Strains of polyhydrolytic aerobic haloalkaliphilic bacteria, isolated from soda solonchak soils. Candidate new species are highlighted in bold (<97% 16S rRNA gene sequence identity). “ +” and “ −” presence or absence of the feature.

Isolate code	Source	Colony morphology			Phylogeny	
	Sample code	Mycelium	Pigment aerial/substrate	Endo-spores	Closest relative	% similarity
***Actinobacteria***						
**DS1**	KUS	+	−	−	*Streptomyces sodiiphilus* (haloalkaliphile)	**97**
**DS7**	BS	+	Gray	−	*Streptomyces sodiiphilus* (haloalkaliphile)	**97**
**DS8**	BS	+	−	−	*Streptomyces sodiiphilus* (haloalkaliphile)	**97**
DS9	BS	+	Gray	−	*Streptomyces alkaliphilus* (haloalkaliphile)	99
DS16	KT	+	−	−	*Streptomyces alkalithermotolerans* (haloalkaliphile)	98
DS31	EWN	+	Gray	−	*Streptomyces* sp. E-070 (haloalkaliphile)	99
DS32	EWN	+	−	−	*Streptomyces* sp. E-070 (haloalkaliphile)	99
**DS34**	MLC	+	Gray	−	*Streptomyces* sp. YIM 80244 (haloalkaliphile)	**97**
DS35	MLC	+	Beige	−	*Streptomyces* sp. E-070 (alkaliphile)	99
DS36	KS	+	Gray	−	*Streptomyces sodiiphilus* YIM 80305 (haloalkaliphile)	99
DS37	KS	+	Gray	−	*Streptomyces alkaliphilus* (haloalkaliphile)	99
DS39	KS	+	-/brown	−	*Streptomyces* sp. E-070 (haloalkaliphile)	99
**DS42**	KS	+	Beige	−	*Streptomyces alkalithermotolerans* (haloalkaliphile)	**97**
DS43	KS	+	Beige	−	*Streptomyces sodiiphilus*	99
DS46	KS	+	Gray	−	*Streptomyces* sp. E-070 (haloalkaliphile)	99
**DS55**	AA	+	−	−	*Streptomyces sodiiphilus* (haloalkaliphile)	**97**
**DS58**	KS	+	−	−	*Streptomyces sodiiphilus* YIM 80305 (haloalkaliphile)	**97**
**DS59**	KS	+	−	−	*Streptomyces sodiiphilus* YIM 80305 (haloalkaliphile)	**97**
DS61	KS	+	Beige	−	*Streptomyces sunnurensis*	98
DS65	AA	+	Gray	−	*Streptomyces alkaliphilus* (haloalkaliphile)	99
**DS70**	AA	+	−	−	*Streptomyces alkalithermophilus* (alkaliphile)	**97**
DS71	AA	+	Gray/red	−	*Streptomyces alkaliphilus* (haloalkaliphile)	99
DS177	KS	−	Gray/viol	−	*Streptomyces alkaliphilus* (haloalkaliphile)	99
DS182	KT	+	Olive	−	*Streptomyces alkaliphilus* (haloalkaliphile)	99
**DS183**	KT	+	−	−	*Streptomyces* sp. E-070 (haloalkaliphile)	**97**
DS2	KUS	+	−	−	*Nocardiopsis exhalans* VTT E-063001	99
DS3	KUS	+	−	−	*Nocardiopsis* sp. YIM 80251 (haloalkaliphile)	99
DS4	KUS	+	−	−	*Nocardiopsis* sp. E-143 (haloalkaliphile)	99
DS10	BS	+	−	−	*Nocardiopsis exhalans* VTT E-063001	99
DS12	KT	+	−	−	*Nocardiopsis* sp. YIM 80129 (haloalkaliphile)	99
DS13	KUS	+	−	−	*Nocardiopsis* sp. E-143 (haloalkaliphile)	99
DS14	KT	+	−	−	*Nocardiopsis* sp. E-143 (haloalkaliphile)	99
DS15	KT	+	−	−	*Nocardiopsis* sp. E-143 (haloalkaliphile)	99
DS17	MS	+	Beige	−	*Nocardiopsis* sp. E-143 (haloalkaliphile)	99
DS18	MS	+	Beige	−	*Nocardiopsis* sp. E-143 (haloalkaliphile)	99
DS19	MS	+	Gray	−	*Nocardiopsis* sp*.* E-143 (haloalkaliphile)	99
DS21	MS	+	Olive	−	*Nocardiopsis* sp. E-143 (haloalkaliphile)	99
DS22	MS	+	−	−	*Nocardiopsis* sp. E-143 (haloalkaliphile)	99
DS23	MS	+	Beige	−	*Nocardiopsis* sp. YIM 80251 (haloalkaliphile)	99
DS24	MS	+	Beige	−	*Nocardiopsis* sp. YIM 80251 (haloalkaliphile)	99
DS25	MS	+	Beige	−	*Nocardiopsis* sp. E-143 (haloalkaliphile)	99
DS26	MS	+	Beige	−	*Nocardiopsis* sp. YIM 80133 (haloalkaliphile)	99
DS27	MS	+	Beige	−	*Nocardiopsis* sp. E-143 (haloalkaliphile)	99
DS28	MS	+	-/brown	−	*Nocardiopsis* sp. YIM 80133 (haloalkaliphile)	99
DS29	MS	+	−	−	*Nocardiopsis* sp. YIM 80133 (haloalkaliphile)	99
DS30	MS	+	−	−	*Nocardiopsis* sp. E-143 (haloalkaliphile)	99
DS38	KS	+	Beige/red	−	*Nocardiopsis* sp. E-143 (haloalkaliphile)	99
DS40	KS	+	Beige	−	*Nocardiopsis* sp. YIM 80129 (haloalkaliphile)	99
DS41	KS	+	Beige	−	*Nocardiopsis* sp*.* AACh2 (haloalkaliphile)	99
DS44	KS	+	−	−	*Nocardiopsis* sp. E-143 (haloalkaliphile)	99
DS45	KS	+	−	−	*Nocardiopsis* sp. YIM 80129 (haloalkaliphile)	100
DS47	AA	+	−	−	*Nocardiopsis alba*	99
DS48	AA	+	−	−	*Nocardiopsis alba*	98
DS49	AA	+	−	−	*Nocardiopsis sinuspersici*	99
DS50	AA	+	−	−	*Nocardiopsis* sp. YIM 80133 (haloalkaliphile)	99
DS51	AA	+	−	−	*Nocardiopsis* sp. E-143 (haloalkaliphile)	99
DS53	AA	+	−	−	*Nocardiopsis* sp. E-143 (haloalkaliphile)	99
DS54	AA	+	-/red	−	*Nocardiopsis* sp. E-143 (haloalkaliphile)	99
DS56	AA	+	−	−	*Nocardiopsis alba*	99
DS57	KS	+	Beige	−	*Nocardiopsis* sp. YIM 80133 (haloalkaliphile)	99
DS62	KS	+	Olive	−	*Nocardiopsis* sp. E-143 (haloalkaliphile)	99
DS63	AA	+	−	−	*Nocardiopsis* sp. E-143 (haloalkaliphile)	99
DS64	AA	+	−	−	*Nocardiopsis* sp. E-143 (haloalkaliphile)	99
DS66	AA	+	−	−	*Nocardiopsis* sp. YIM 80130 (haloalkaliphile)	99
DS67	AA	+	−	−	*Nocardiopsis* sp. AACh2 (haloalkaliphile)	99
DS68	AA	+	−	−	*Nocardiopsis* sp. YIM 80130 (haloalkaliphile)	99
DS69	AA	+	−	−	*Nocardiopsis* sp. E-143 (haloalkaliphile)	99
DS73	KUS	+	−	−	*Nocardiopsis* sp*.* AACh2 (haloalkaliphile)	99
DS74	KUS	+	−	−	*Nocardiopsis* sp. AACh2 (haloalkaliphile)	99
DS75	KUS	+	−	−	*Nocardiopsis* sp. E-143 (haloalkaliphile)	99
DS76	KUS	+	−	−	*Nocardiopsis* sp. E-143 (haloalkaliphile)	99
DS78	KUS	+	−	−	*Nocardiopsis* sp. YIM 80130 (haloalkaliphile)	99
DS79	KUS	+	−	−	*Nocardiopsis* sp. AACh2 (haloalkaliphile)	99
DS174	KS	−	-/red		*Nocardiopsis* sp. E-143 (haloalkaliphile)	99
DS175	KS	−	−	−	*Nocardiopsis* sp. E-143 (haloalkaliphile)	99
DS176	KS	−	−	−	*Nocardiopsis* sp. E-143 (haloalkaliphile)	99
DS178	KS	−	−	−	*Nocardiopsis* sp. YIM 80034 (haloalkaliphile)	100
DS180	KUS	+	Reddish	−	*Nocardiopsis ganjiahuensis* (haloalkaliphile)	100
DS181	KUS	+	−	−	*Nocardiopsis* sp. AACh2 (haloalkaliphile)	99
**DS20**	MS	+	−	−	***Glycomycetaceae*(halophiles)**	**92**
**DS33**	EWN	+	Pink	−	***Salinispora arenicola*** NPS11684	**94**
DS60	KS	+	−	−	*Isoptericola halotolerans*	99
DS82	KT	−	Yellow	+	*Isoptericola halotolerans*	99
DS88	KS	−	Yellow	−	*Isoptericola halotolerans*	99
DS91	KT	−	Yellow	−	*Isoptericola halotolerans*	99
DS92	KT	−	Yellow	−	*Isoptericola halotolerans*	99
DS97	MS	−	Yellow	−	*Isoptericola halotolerans*	99
DS99	MS	−	−	−	*Isoptericola halotolerans*	99
DS111	MS	−	Yellow	−	*Isoptericola halotolerans*	98
DS164	KS	−	Yellow	−	*Isoptericola halotolerans*	99
DS149	MS	−	Orange	−	*Nesterenkonia xinjiangensis*	100
DS11	KUS	−	−	−	*Nitriliruptor alkaliphilus* (haloalkaliphile)	98
***Bacilli***						
DS6	BS	+	−	+	*Bacillus horikoshii* (alkaliphile)	100
DS72	KUS	−	−	+	*Bacillus* sp. *E-141* (haloalkaliphile)	99
DS81	KT	−	−	+	*Bacillus okhensis* (haloalkalitolerant)	99
DS83	KT	−	−	+	*Bacillus* sp. ABCh1 (haloalkaliphile)	98
DS84	KT	−	Yellow	+	*Bacillus cellulolyticus* (alkaliphile)	99
DS85	KT	−	−	+	*Bacillus cellulolyticus* (alkaliphile)	99
DS86	KT	−	Cream	+	*Bacillus pseudofirmus (* alkaliphile)	100
DS87	KT	−	−	+	*Bacillus polygoni* (haloalkaliphile)	99
DS89	KS	−	−	+	*Bacillus daliensis* (haloalkaliphile)	99
DS90	KT	−	−	+	*Bacillus halodurans (* haloalkalitolerant)	100
DS93	KT	−	−	+	*Bacillus cellulolyticus* (alkaliphile)	100
DS94	KT	−	−	+	*Bacillus vedderi* (alkaliphile)	98
DS95	KT	−	−	+	*Bacillus akibai* (alkaliphile)	98
DS96	MS	−	Orange	−	*Bacillus halodurans* (haloalkaliphile)	99
DS100	MS	−	Orange	+	*Bacillus daliensis* (haloalkaliphile)	98
DS101	MS	−	−	+	*Bacillus akibai* (alkaliphile)	99
DS102	MS	−	−	+	*Bacillus alkalisediminis* (haloalkaliphile)	98
DS103	MS	−	−	+	*Bacillus akibai* (alkaliphile)	99
DS104	MS	−	−	+	*Bacillus alkalisediminis* (haloalkaliphile)	98
DS105	MS	−	−	+	*Bacillus akibai* (alkaliphile)	99
DS106	MS	−	−	+	*Bacillus alkalisediminis* (haloalkaliphile)	98
DS107	MS	−	−	+	*Bacillus akibai* (alkaliphile)	99
DS108	MS	−	−	+	*Bacillus alkalisediminis* (haloalkaliphile)	98
DS109	MS	−	−	+	*Bacillus alkalisediminis* (haloalkaliphile)	98
DS110	MS	−	−	+	*Bacillus akibai* (alkaliphile)	99
DS112	MS	−	−	+	*Bacillus pseudofirmus* (alkaliphile)	99
DS113	KS	−	Orange	−	*Bacillus daliensis* (haloalkaliphile)	99
**DS114**	KT	−	−	+	*Bacillus bogoriensis* (haloalkaliphile)	**97**
DS116	KT	−	−	+	*Bacillus* sp. Z24-11 (haloalkaliphile)	100
DS118	KT	−	−	+	*Bacillus polygoni* (alkaliphile)	99
DS119	KT	−	−	+	*Bacillus pseudofirmus* (alkaliphile)	100
DS120	KT	−	−	+	*Bacillus pseudofirmus* (alkaliphile)	99
DS121	KT	−	−	+	*Bacillus pseudofirmus* (alkaliphile)	99
DS122	KT	−	Cream	+	*Bacillus pseudofirmus* (alkaliphile)	98
DS126	BS	−	−	+	*Bacillus pseudofirmus* (alkaliphile)	99
DS127	BS	−	Orange	+	*Bacillus pseudofirmus* (alkaliphile)	99
DS128	BS	−	Orange	+	*Bacillus pseudofirmus* (alkaliphile)	99
DS129	BS	−	−	+	*Bacillus pseudofirmus* (alkaliphile)	99
DS131	BS	−	Orange	−	*Bacillus pseudofirmus* (alkaliphile)	100
DS132	KT	−	Cream	+	*Bacillus polygoni* (haloalkaliphile)	99
DS133	KT	−	−	+	*Bacillus halodurans* (haloalkaliphile)	100
DS134	KT	−	Cream	+	*Bacillus clarkii* (alkaliphile)	99
DS135	KT	−	−	+	*Bacillus polygoni* (haloalkaliphile)	99
DS136	KT	−	Cream	+	*Bacillus* sp. Z24-11 (haloalkaliphile)	99
DS137	KT	−	−	+	*Bacillus pseudofirmus* (alkaliphile)	99
DS138	KT	−	−	+	*Bacillus* sp. Z24-11 (haloalkaliphile)	99
DS139	KT	−	−	+	*Bacillus polygoni* (haloalkaliphile)	100
DS140	KT	−	−	+	*Bacillus alkalisediminis* (haloalkaliphile)	99
DS141	KT	−	Yellow	+	*Bacillus alkalinitrilicus* (haloalkaliphile)	99
DS142	KT	−	−	+	*Bacillus alkalinitrilicus* (haloalkaliphile)	99
**DS143**	KT	−	−	+	***Bacillus mannanilyticus*** (alkaliphile)	**96**
DS144	MS	−	−	+	*Bacillus pseudofirmus* (alkaliphile)	99
DS148	MS	−	−	+	*Bacillus alkalinitrilicus* (haloalkaliphile)	99
DS150	MS	−	Orange	+	*Bacillus daliensis* (haloalkaliphile)	98
DS151	MS	−	−	+	*Bacillus halodurans* (haloalkaliphile)	100
DS152	MS	−	−	+	*Bacillus horikoshii* (alkaliphile)	99
DS153	MS	−	−	+	*Bacillus pseudofirmus* (alkaliphile)	99
DS155	MS	−	−	+	*Bacillus pseudofirmus* (alkaliphile)	99
DS158	MS	−	−	+	*Bacillus pseudofirmus* (alkaliphile)	99
DS159	MS	−	−	+	*Bacillus akibai* (alkaliphile)	99
DS160	KS	−	Yellow	+	*Bacillus horikoshii* (alkaliphile)	99
DS161	KS	−	−	+	*Bacillus horikoshii* (alkaliphile)	99
DS163	KS	−	−	+	*Bacillus pseudofirmus* (alkaliphile)	100
DS165	KS	−	−	+	*Bacillus pseudofirmus* (alkaliphile)	99
DS166	KS	−	−	+	*Bacillus pseudofirmus* (alkaliphile)	99
DS168	KS	−	−	+	*Bacillus pseudofirmus* (alkaliphile)	99
DS169	KS	−	−	+	*Bacillus pseudofirmus* (alkaliphile)	99
DS172	KS	−	−	+	*Bacillus pseudofirmus* (alkaliphile)	99
DS184	KT	−	−	+	*Bacillus halodurans* (haloalkaliphile)	100
**DS117**	KT	−	Orange	−	*Anaerobacillus alkalidiazotrophicus* (haloalkaliphile)	**97**
**DS123**	KT	−	−	+	*Anaerobacillus alkalidiazotrophicus* (haloalkaliphile)	**97**
***Gammaproteobacteria***						
DS115	KUS	−	−	−	*Alkalimonas amylolytica* (haloalkaliphile)	99
DS125	BS	−	−	−	*Alkalimonas collagenimarina* (haloalkaliphile)	99
DS130	BS	−	−	−	*Alkalimonas amylolytica* (haloalkaliphile)	99
DS154	MS	−	Greenish	−	*Alkalimonas amylolytica* (haloalkaliphile)	99
DS124	BS	−	−	−	*Aliidiomarina maris*	99
DS145	MS	−	−	−	*Aliidiomarina soli* (haloalkaliphile)	99
DS146	MS	−	−	−	*Aliidiomarina soli* (haloalkaliphile)	99
DS156	MS	−	−	−	*Aliidiomarina soli* (haloalkaliphile)	99
DS157	MS	−	−	−	*Aliidiomarina soli* (haloalkaliphile)	99
DS167	KS	−	−	−	*Aliidiomarina soli* (haloalkaliphile)	99
DS179	KS	−	−	−	*Aliidiomarina soli* (haloalkaliphile)	98
**DS162**	KS	−	Yellow	−	*Xanthomonadaceae* ML-122 (haloalkaliphile) ***Rehaibacterium terrae***	**97** **95**
DS170	KS	−	−	−	*Xanthomonadaceae* ML-122 (haloalkaliphile)	99
**DS171**	KS	−	−	−	***Lysobacter*spp**.	**96**
DS173	KS	−	Yellow	−	*Xanthomonadaceae* ML-122 (haloalkaliphile) ***Lysobacter*spp.**	99 **95**
**DS147**	MS	−	−	−	*Xanthomonadaceae* ML-122 (haloalkaliphile) ***Lysobacter*spp.**	99 **95**

The general phylogenetic distribution of the isolates is shown on a Krona diagram, obtained in the course of SILVAngs analysis (Fig. 1) and in the sample-dependent taxa clustering (Table S1). The *Actinobacteria* were mostly represented by two genera—*Nocardiopsis* and *Streptomyces*, and they were closely related to halotolerant alkaliphilic strains and species of these two genera found previously in haloalkaline habitats, in particular in Kenyan and Chinese soda lakes and saline alkaline soils (Grant & Jones, 2016). The relatively low diversity within the otherwise extremely diverse genera of these *Actinobacteria* indicates that haloalkaline conditions are rather selective for a few highly adapted species. Only two isolates from this group were distantly related to known species. One strain might represent a new genus in the *Micromonosporacea* with a closest relative from the genus *Salinispora*, while the second isolate is a distant member in the family *Glycomycetaceae* (Figs. S1A and S1B, respectively).

**Table 3:** Polymer hydrolysis and utilization by aerobic haloalkaliphiles from soda soils.

Strain code	Enriched with:	CMC		Xylane			Starch		Casein		Olive oil	
		Activity		Growth	Activity		Growth/activity		Growth/activity		Activity	
		*ϕ* col	*ϕ* zone		*ϕ* col	*ϕ* zone	*ϕ* col	*ϕ* zone	*ϕ* col	*ϕ* zone	*ϕ* col	*ϕ* zone
Ds1	**CMC**	2	−		−		3	19	4	**30**	4	8
Ds11		4	16		−		5	20	−		−	
Ds2		7	20	+	6	**30**	8	22	7	32	10	12
Ds3		8	24	+	4	**22**	4	25	6	30	8	11
Ds4		2	18	+	6	**27**	8	25	8	30	10	12
Ds180		7	19		6	**32**	8	28	9	30	8	13
Ds181		7	23		6	22	5	24	10	**35**	9	13
Ds6		1	12		−		3	20	4	25	−	
Ds7		2	14	Weak	2	**18**	3	24	5	22	8	13
Ds8^a^		2	14	+	2	15	4	20	3	20	−	
Ds9		4	12	+	5	**25**	5	20	5	**35**	10	13
Ds10		6	17	+	5	**28**	7	24	10	30	15	17
Ds182^c^		3	16		3	**24**	3	28	5	**30**	5	8
Ds183		2	10		3	12	3	20	5	28	−	
Ds12		6	18	+	5	**25**	7	24	10	25	12	14
Ds13		7	19	+	5	**26**	7	25	6	25	12	14
Ds14		5	17	+	5	**30**	9	25	5	25	12	14
Ds15		5	20		5	−	3	17	2	−	5	7
Ds16^a^		5	20	+	5	22	4	15	2	23	8	13
Ds17		6	21	+	6	**28**	8	24	6	22	10	12
Ds18		5	14	+	5	**25**	7	22	5	24	7	9
Ds19		7	16		3	−	7	25	4	**28**	10	12
Ds20		5	14		4	−	3	−	4	18	−	
Ds21		7	17	+	2	18	9	32	5	27	−	
Ds22		4	13		7	**-**	4	15	4	**25**	2	**10**
Ds23		6	16	+	7	**26**	6	30	5	20	10	12
Ds24		4	14	+	5	18	6	30	4	**28**	8	10
Ds25		4	12	+	7	**30**	9	27	5	22	10	12
Ds26		2	13		2	−	2	10	3	**25**	−	
Ds27		5	15	+	7	**26**	10	26	4	**25**	10	11
Ds28		4	14	+	6	21	8	15	5	25	7	10
Ds29		2	9		3	−	4	9	3	24	−	
Ds30		6	17		7	**26**	9	28	5	20	12	14
Ds31		8	17		2	**25**	5	23	6	22	10	13
Ds32		4	17		3	**23**	6	22	2	20	5	9
Ds33^b^		5	20	+	2	**28**	2	16	2	20	−	
Ds34		3	12		6	**40**	5	30	5	23	6	10
Ds35		4	18	Weak	4	20	3	20	6	22	**5**	**13**
Ds36		3	22	+	4	**23**	4	30	4	25	7	12
Ds37		3	10	+	3	12	6	25	6	28	6	9
Ds38		5	15	+	4	**25**	7	24	6	28	13	14
Ds39		2	12		3	−	6	25	2	12	10	10
Ds40		5	15	+	7	**23**	7	27	4	23	9	11
Ds41		6	16	+	7	**23**	5	23	5	27	9	11
Ds42		2	14		2	−	2	3	3	**27**	7	10
Ds43^c^		2	24		2	14	4	28	3	**32**	6	10
Ds44		5	20	+	7	**30**	8	27	5	22	9	12
Ds45		3	15	+	5	**30**	7	25	4	20	−	
Ds46		2	10	+	2	**20**	4	22	3	20	8	10
Ds47		5	21	+	5	**23**	7	27	8	28	10	14
Ds48		3	15	+	4	17	4	20	4	20	8	10
Ds49		2	13	+	4	17	5	23	10	**35**	8	10
Ds50		3	15		7	**26**	5	14	6	17	8	10
Ds51		3	15	+	5	**23**	7	30	8	30	10	13
Ds53		3	18		−		2	20	−		−	
Ds54		2	12	+	6	**24**	9	29	8	30	10	13
Ds55		4	15		1	**23**	4	22	4	25	5	7
Ds56		4	17	+	5	**23**	9	29	7	26	7	9
Ds81		2	10		2	**24**	5	24	–		–	
Ds82		3	21	+	4	**24**	6	28	5	**30**	7	8
Ds83		2	15	Weak	2	16	5	32	3	−	−	
Ds84		3	19	Weak	3	15	4	24	4	20	−	
Ds85		3	14	Weak	4	15	5	25	3	20	−	
Ds86		1.5	**20**		2	−	4	28	5	**30**	−	
Ds87		2	16	Weak	2	**21**	4	17	3	12	−	
Ds88		4	22		4	20	6	22	3	20	−	
Ds89		3	12	+	2	**23**	3	25	2	−	−	
Ds90		4	15	+	3	**27**	4	25	3	−	−	
Ds91^c^		5	20	**++**	3	**29**	5	24	7	20	8	**15**
Ds92^c^		5	23	+	6	**28**	7	32	7	30	3	6
Ds93		3	18		3	15	4	10	7	15	3	7
Ds94		2	14		2	−	4	9	3	15	3	5
Ds95		2	8	+	4	**30**	4	23	4	11	−	
Ds96		2	20	++	3	**26**	5	24	3	10	−	
Ds97		3	22		5	14	5	28	4	15	−	
Ds98		5	23	+	6	**24**	5	25	3	−	11	14
Ds99		2	21		3	14	4	20	2	8	−	
Ds100		2	24	+	2	**27**	3	29	1	−	−	
Ds101		3	22	+	3	**22**	6	32	1	12	−	
Ds102		5	23		3	8	5	18	2	−	−	
Ds103		3	28	+	4	**22**	5	28	1	10	−	
Ds104		2	18		3	10	4	19	4	12	−	
Ds105		3	27	+	3	**22**	**5**	**34**	4	21	−	
Ds106		3	25		4	11	6	18	4	20	−	
Ds107		3	27	Weak	3	18	4	28	4	20	−	
Ds108		3	28		2	−	5	18	5	23	−	
Ds109		2	25		4	11	4	18	5	22	−	
Ds110		2	27	+	3	**20**	4	**35**	7	25	−	
Ds111^c^	3	26	+		4	**20**	3	25	5	17	7	7
Ds112		3	25	+	4	21	4	25	7	20	−	
Ds113		2	13	Weak	2	15	4	23	2	−	−	
Ds184		5	12		9	**34**	6	25	4	25	10	**16**
Ds57	**Casein**	5	20	+	4	19	8	26	5	**28**	12	14
Ds58		−		+	4	17	−		4	22	−	
Ds59		4	17		−	−			2	16	−
Ds60		3	0	Weak	4	23	5	24	5	17	−	
Ds61		4	0		−		3	10	2	20	−	
Ds62		1	7		2	14	3	24	3	20	−	
Ds114		−		+	4	27	5	28	4	20	−	
Ds115		−			−		5	30	4	20	4	10
Ds116		−			2	17	4	16	2	15	−	
Ds117		−			2	10	5	20	3	20	−	
Ds118		−			3	12		−	2	18	6	11
Ds119		−			−		3	**30**	4	18	−	
Ds120		−			2	10	4	30	2	24	−	
Ds121		−		+	6	29	3	**30**	3	24	−	
Ds122		−			5	−	−		2	22	−	
Ds123		−		+	4	17	4	15	4	20	−	
Ds124		−				−	−		4	22	−	
Ds125		−			−		5	20	5	24	−	
Ds126		−			−		5	25	2	18	−	
Ds127		−			−		4	28	2	12	−	
Ds128		−			−		5	32	2	22	−	
Ds129		−		+	3	13	4	32	3	20	−	
Ds130		−			−		3	**40**	5	23	4	8
Ds131		−		Weak	2	10	3	**33**	4	15	−	
Ds132		−			3	14	−		3	12	−	
Ds133		4	20		3	**20**	7	25	5	15	−	
Ds134		−		Weak	2	19	−		2	20	−	
Ds135		−			2	15	−		3	15	−	
Ds136		−			−		4	20	3	17	−	
Ds137		−			−		5	29	3	15	−	
Ds138		−			−		4	28	2	14	−	
Ds139		−			3	18	−		3	14	−	
Ds140		−		Weak	2	11	4	**33**	5	22	−	
Ds141		−			−		−		2	14	−	
Ds142		−			−		−		3	17	5	15
Ds143		−			−		−		3	22	−	
Ds144		−			−		5	30	5	23	−	
Ds145		−			−		−		5	19	−	
Ds146		−			−		−		5	24	−	
Ds147		−			−		−		4	22	−	
Ds148		−			8	−	−		3	20	−	
Ds149		−			−		3	28	3	20		w
Ds150		−		+ +	4	**31**	5	25	3	14	−	
Ds151		4	23	Weak	3	17	7	25	4	24	−	
Ds152		−			−		3	20	3	23	−	
Ds53		−			−		3	23	2	20	−	
Ds154		−			−		4	**30**	6	20	1	5
Ds155		5	17		3	12	6	28	3	15	9	11
Ds156		−			−		−		5	15	−	
Ds157		−			−		−		5	17	−	
Ds158		−			5	9	5	29	4	10	−	
Ds159		5	28	+	5	**30**	5	30	2	12	−	
Ds160		−			2	0	5	30	3	22	−	
Ds161		−					4	25	2	**28**	−	
Ds162		−			3	15	−		1	17	−	
Ds163		−					3	25	2	25	−	
Ds164		3	22	+	3	18	6	26	4	25	−	
Ds165		−			−		3	28	3	20	−	
Ds166		−			−		5	26	3	15	−	
Ds167		−			−		5	27	4	20	−	
Ds168		−			−		5	26	3	20	−	
Ds169		−			−		5	30	2	22	−	
Ds170	**Keratin**	−			−		−		4	20	−	
Ds171		−			−		−		5	23	−	
Ds172		−			−		5	32	2	20	−	
Ds173		−			−		−		3	18	−	
Ds174		3	20		−		5	25	9	25	12	14
Ds175		−			−		3	**30**	8	30	10	12
Ds176		−		+	8	**35**	9	25	9	30	10	12
Ds177		1	7	+	2	24	5	19	4	25	7	9
Ds178		5	22		3	−	7	22	8	30	9	10
Ds179		5	14	+	8	**34**	8	25	10	30	10	13
Ds63	**Starch**	2	10	+	5	24	7	26	7	25	10	12
Ds64		5	13	+	6	28	8	24	10	33	11	16
Ds65		2	13		4	**27**	5	24	6	30	6	9
Ds66		2	12		3	−	5	20	5	25	10	15
Ds67		−		+	5	23	5	28	3	22	6	12
Ds68		3	10	+	6	25	6	25	5	25	12	15
Ds69		5	15		3	20	6	28	6	29	11	15
Ds70		−			−		−		2	15	2	6
Ds71		1	8	+	4	20	5	25	−		8	11
Ds72		5	12		4	30		−	10	30	8	14
Ds73		2	8	+	5	24	6	20	10	32	8	13
Ds74	**Olive oil**	3	13	+	8	18	7	30	10	32	10	14
Ds75		8	20	+	6	**35**	10	30	11	30	10	14
Ds76		5	18	+	7	28	7	25	8	30	−	
Ds78		4	10		2	−	6	20	5	12	12	13
Ds79		2	13	+	4	21	6	24	8	15	6	9

**Notes.**

CMCase-4 d, Xylanase, protease, amylase-3 d; lipase-10d; amorphous cellulose and chitin-30 d; *ϕ* col-colony diameter, mm; *ϕ* zone-hydrolysis zone diameter, mm. Highlights: on the basis of activity to colony diameter ratio: highly active-in bold. Mean values from two biological replicates.

a Positive on amorphous cellulose.

b Growth on amorphous cellulose.

c Growth on amorphous chitin.

Same low genetic diversity was also observed in the second largest group represented by the genus *Bacillus*. Most of the isolates were closely related to the known alkaliphilic (*B. pseudofirmus*, *B. horokoshii* and *B. akibai*), or haloalkaliphilic (*B. halodurans*, *B. daliensis*, and *B. alkalisediminis*) species. The only exception was a single isolate only distantly related (95% sequence similarity) to *B. mannanilyticus*—a low salt-tolerant alkaliphilic species producing beta-mannanase (Akino, Nakamura & Horikoshi, 1987; Nogi, Takami & Horikoshi, 2005) (Fig. S1C).

A relatively minor group of isolates enriched with proteins belonged to the proteobacterial class *Gammaproteobacteria*. A subgroup of three isolates was closely related (99% sequence similarity) to species of the genus *Alkalimonas*, a known amylolytic haloalkaliphile (Ma et al., 2004). Four isolates were closely related to a haloalkaliphilic member of the genus *Aliidiomarina*, *A. soli*, isolated from a soda soil in Inner Mongolia (Xu & Wu, 2017). The third gammaproteobacterial subgroup is represented by 4 proteolytic strains distantly related to organisms in the genus *Lysobacter* in the *Xanthomonadaceae* (95–96% sequence similarity). Three out of four strains of this subgroup clustered with an undescribed haloalkaliphilic isolate from Mono Lake (ML-122, 99% similarity), while the fourth strain was distant (96% similarity to ML-122). Therefore, this subgroup probably consists of two novel species and together with the Mono Lake strain ML-122 might represent a new genus in the family *Xanthomonadacea* (Fig. S1D).

Finally, a significant group of actinobacteria with strong polyhydrolytic potential belonged to the *Cellulomonas/Isoptericola* clad within the family *Promicromonosporaceae* (Fig. S1E). The *Cellulomonas* species are known for their cellulolytic activity and include a haloalkaliphilic isolate from a Kenyan soda lake (Jones et al., 2005), while the genus *Isoptericola* mostly include halotolarant representatives, although the described neutrophic species apparently have only a limited hydrolytic activity (Schumann & Stackebrandt, 2014).

**Table 4:** Influence of pH on growth and endoglucanase activity of soda solonchak alkaliphiles: average profiles estimated from individual results for eight isolates: actinomycetes-*Nocardiopsis* DS50, 51; *Streptomyces* DS8,9; *Bacillus*: DS85, 100, 101, 102.

pH	% of maximum			
	*Actinomycetes*		*Bacillus* ACB	
	Growth	Activity	Growth	Activity
5	0		0	
6	20–70	30–70	0	
7	40–100	70–100	0–10	0–40
8	80–100	90–100	20–60	40–100
9	90–100	90–100	70–100	90–100
10	80–100	90–100	100	90–100
10.5	40–90	70–100	80–100	100
11	10–40	40–80	30–70	50–90

**Notes.**

Solid medium 0.6 M total Na^+^ buffered with: pH 5–8-0.1 M HEPES/NaCl/NaHCO_3_; pH 8–11-NaHCO_3_/Na_2_CO_3_. Substrate: 0.1% CMC + yeast extract 0.2 g/l. Growth and activity were estimated by the diameter of colony and zone of hydrolysis, respectively, after four days of plate incubation at 30 °C.

### Hydrolytic spectra of the soda soil isolates

Most of the actinobacteria and bacilli isolates enriched with CMC or starch, were polyhydrolytic, being able to degrade all tested polymers, except for the insoluble native cellulose and chitin (Table 3). Only three actinobacterial isolates showed the ability to hydrolyse amorphous cellulose on the plate assay and only one of the three (DS33), a relative of *Salinispora*, was actually capable of growth with cellulose as substrate. Six isolates showed a potential to grow with amorphous chitin (Table 3). On the other hand, most of the endo-glucanase and endoxylanase positive actinobacteria and bacilli isolates utilized beech xylan as the growth substrate, which indicates that they are rather specialized in the mineralization of soluble hemicelluloses.

The isolates enriched with proteins belonged to the *Gammaproteobacteria* and *Firmicutes.* All of them, as expected, showed highest hydrolytic potential against casein, and many of them did not have endoglucanase, endoxylanase or lipase activities (Table 3). So, they can be considered as dedicated proteolytics. Indeed, proteolytics are the most well-studied group of alkaliphilic hydrolytics.

For the pH profiling, four strains from actinomycetes and from bacilli were selected for test on solid medium containing 0.6 M total Na^+^ with CMC + yeast extract as substrate. The solid medium is not optimal for the profiling but it was chosen for two reasons: (1) the mycelium-forming actinomycetes do not grow homogenously in liquid media and their growth is often estimated by radial colony increase; (2) test on solid medium permitted simultaneous estimation of both growth and endoglucanase activity. The results (Table 4) demonstrated that the tested actinomycetes are facultative moderate alkaliphiles, while the bacilli isolates are obligate alkaliphiles. The endoglucanase activity of both groups had a very broad pH range from six to 11 with an optimum for actinomycetes from eight to 10 and for the bacilli from nine to 10.5.

Overall, the results of this study demonstrated that saline alkaline soils represent a potentially valuable resource of aerobic haloalkaliphilic bacteria capable of producing multiple alkalistable hydrolytic enzymes. Most of the haloalkaliphilic polyhydrolytic isolates belong to *Actinobacteria* (genera *Streptomyces* and *Nocardiopsis*) and the genus *Bacillus*. We consider the actual capability of a large proportion of the soda soil aerobic haloalkaliphilic isolates to utilize xylan and starch as growth substrates as one of the principal findings of this extended screening. Such organisms definitely represent an interesting object for further investigation of their haloalkalistable hydrolases, particularly with a potential for application in laundry detergent production.

##  Supplemental Information

10.7717/peerj.3796/supp-1 Supplemental Information 1 Supplemental Tables S1–S2, Supplemental Figure S1 Table S1. SILVAngs Project Summary and Project Settings.
Table S2. Site-specific taxonomic distribution of isolates different locations.
Figure S1. Unrooted 16S rRNA gene sequence Maximum likelihood phylogenetic trees of the haloalkaliphilic isolates, possibly representing novel taxa, and its nearest relatives. All positions with less than 95 % site coverage were eliminated. There were a total of 1,338 (A), 1,394 (B), 1,442 (C), 1,373 (D) and 1,103 (E) positions in the final datasets. The tree is drawn to scale, with branch lengths measured in the number of substitutions per site with corrections, associated with the model. Bootstrap values as percentages of 1,000 repetitions are shown next to the branches. Type strains of validly published species are underlined. Genbank numbers are indicated at the beginning of each sequence designation.

## Additional Information and Declarations

### DNA Deposition

The following information was supplied regarding the deposition of DNA sequences: GenBank numbers: KY775645 –KY775672.

### Data Availability

The following information was supplied regarding data availability: The strains are in the laboratory collection, and the 16S sequences were deposited to Genbank under accession numbers KY775645 –KY775672.
