# Supplementary material for: Diversity of cultivated aerobic poly-hydrolytic bacteria in saline alkaline soils
Source: PeerJ. 2017 Sep 25;5:e3796. doi: 10.7717/peerj.3796 (PMC5619242; doi:10.7717/peerj.3796)
Supplement: Supplemental Information 1 — Table S1. SILVAngs Project Summary and Project Settings. Table S2. Site-specific taxonomic distribution of isolates different locations. Figure S1. Unrooted 16S rRNA gene sequence Maximum likelihood phylogenetic trees of the haloalkaliphilic isolates, possibly representing novel taxa, and its nearest relatives. All positions with less than 95 % site coverage were eliminated. There were a total of 1,338 (A), 1,394 (B), 1,442 (C), 1,373 (D) and 1,103 (E) positions in the final datasets. The tree is drawn to scale, with branch lengths measured in the number of substitutions per site with corrections, associated with the model. Bootstrap values as percentages of 1,000 repetitions are shown next to the branches. Type strains of validly published species are underlined. Genbank numbers are indicated at the beginning of each sequence designation. [file peerj-05-3796-s001.pdf]

Supplementary Table S1. SILVAngs Project Summary and Project Settings.

**Project Summary**	
Project Name:	DS collection
Porject Description:	Default configuration
Sequence Type:	SSU
Number of Samples:	1
Number of Sequences:	179
Number of Rejected Sequences:	0 (0.00%)
**Raw Sequence Information**	
Min. Length:	441
Avg. Length:	1101
Max. Length:	1497
**Aligned Sequence Information**	
Min. Length:	441
Avg. Length:	1101
Max. Length:	1497
**Clustering information**	
Number of OTUs:	45 (25.14 %)
Number of Clustered Sequences:	95 (53.07 %)
Number of Replicates:	39 (21.79 %)
Classification Information	
Number of Classified Sequences:	179 (100.00 %)
Number of "No Relative":	0 (0.00 %)
**Project Settings**	
SINA Version:	SINA v1.2.10 for ARB SVN (revision 21008)
Min Align. Identity (%):	50
Min Align. Score:	40
Min Basepair Score (%):	30
**Quality Control**	
Min. Sequence Quality (%):	30
Min. Length (aligned nuc.):	50
Max. Ambiguities (%):	2
Max. Homopolymers (%):	2
**Clustering**	
CD-Hit Version:	3.1.2
Min. OTU Identity (%):	98
**Classification**	
BLAST Version:	2.2.30+
Reference:	SILVA
Reference Version:	128
Similarity (%):	93

Suppl. Table S2. Site-specific taxonomic distribution of isolates different locations

Group	Armenia	s-w Siberia		s-e Siberia	n-e Monglolia	Kenya-Tanzania	Egypt	California
	**AA**	**KS**	**BS**	**KUS**	**MS**	**KT**	**EWN**	**MLC**
Actinobacteria								
Nocardiopsis alba	4							
Nocardiopsis YIM80133	3	4		2	5	1		
Nocardiopsis E-143	6	6		4	8	3		
Nocardiopsis AACh2	1	1		4				
Nocardiopsis exhalans			1	1				
Streptomyces sodiiphilus	1	4	3	1				
Streptomyces alkaliphilus	2	2				1		
Streptomyces E-070		2				1	2	1
Streptomyces alkalithermotolerans	1	1				1		
Streptomyces YIM80244						1		1
Isoptericola halotolerance		3			3	2		
Bacillales								
B. pseudofirmus		6	5		5	7		
B. akibai					6	1		
B. halodurans					3	2		
B. horikoshii		2	1			2		
B. polygonii						4		
B. cellulolyticus						3		
Bacillus daliensis		2			2			
Bacillus alkalisediminis					5			
B. vedderii						1		
B. bogoriensis						1		
B. clarkii						1		
Bacillus alkalinitrilicus					1			
Bacillus E-141				1				
Anaerobacillus alkalidiazotrophicus						2		
Gammaproteobacteria								
Aliidiomarina			1		4			
Alkalimonas amylolytica		2	2	1	1			
Xanthomonadaceae		3			1			

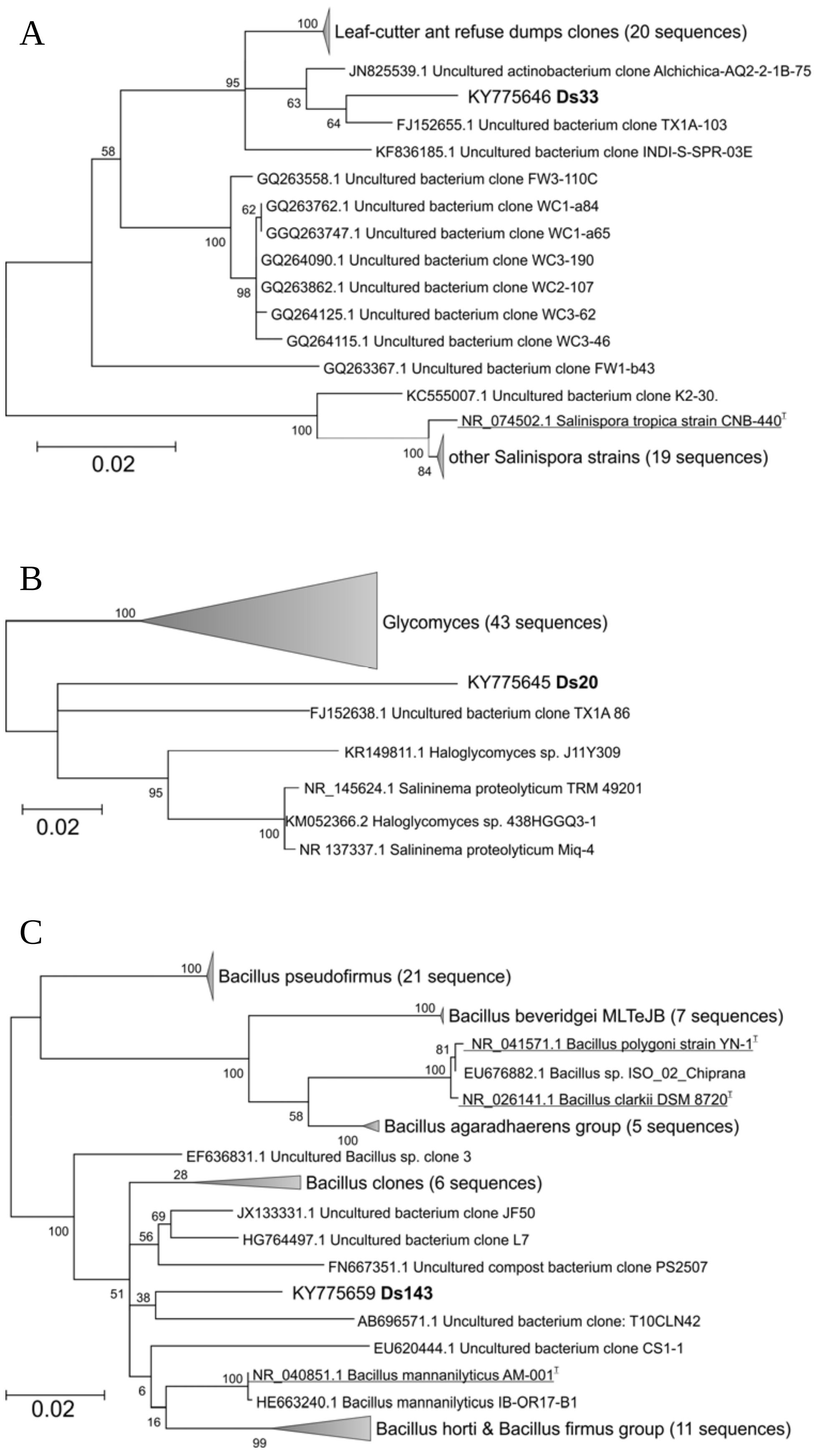
A
Leaf-cutter ant refuse dumps clones (20 sequences)
JN825539.1 Uncultured actinobacterium clone Alchichica-AQ2-2-1B-75
KY775646 **Ds33**
FJ152655.1 Uncultured bacterium clone TX1A-103
KF836185.1 Uncultured bacterium clone INDI-S-SPR-03E
GQ263558.1 Uncultured bacterium clone FW3-110C
GQ263762.1 Uncultured bacterium clone WC1-a84
GGQ263747.1 Uncultured bacterium clone WC1-a65
GQ264090.1 Uncultured bacterium clone WC3-190
GQ263862.1 Uncultured bacterium clone WC2-107
GQ264125.1 Uncultured bacterium clone WC3-62
GQ264115.1 Uncultured bacterium clone WC3-46
GQ263367.1 Uncultured bacterium clone FW1-b43
KC555007.1 Uncultured bacterium clone K2-30.
NR_074502.1 Salinispora tropica strain CNB-440T
other Salinispora strains (19 sequences)
0.02
B
Glycomyces (43 sequences)
KY775645 **Ds20**
FJ152638.1 Uncultured bacterium clone TX1A 86
KR149811.1 Haloglycomyces sp. J11Y309
NR_145624.1 Salininema proteolyticum TRM 49201
KM052366.2 Haloglycomyces sp. 438HGGQ3-1
NR 137337.1 Salininema proteolyticum Miq-4
0.02
C
Bacillus pseudofirmus (21 sequence)
Bacillus beveridgei MLTeJB (7 sequences)
NR_041571.1 Bacillus polygoni strain YN-1T
EU676882.1 Bacillus sp. ISO_02_Chiprana
NR_026141.1 Bacillus clarkii DSM 8720T
Bacillus agaradhaerens group (5 sequences)
EF636831.1 Uncultured Bacillus sp. clone 3
Bacillus clones (6 sequences)
JX133331.1 Uncultured bacterium clone JF50
HG764497.1 Uncultured bacterium clone L7
FN667351.1 Uncultured compost bacterium clone PS2507
KY775659 **Ds143**
AB696571.1 Uncultured bacterium clone: T10CLN42
EU620444.1 Uncultured bacterium clone CS1-1
NR_040851.1 Bacillus mannanilyticus AM-001T
HE663240.1 Bacillus mannanilyticus IB-OR17-B1
Bacillus horti & Bacillus firmus group (11 sequences)
0.02

D

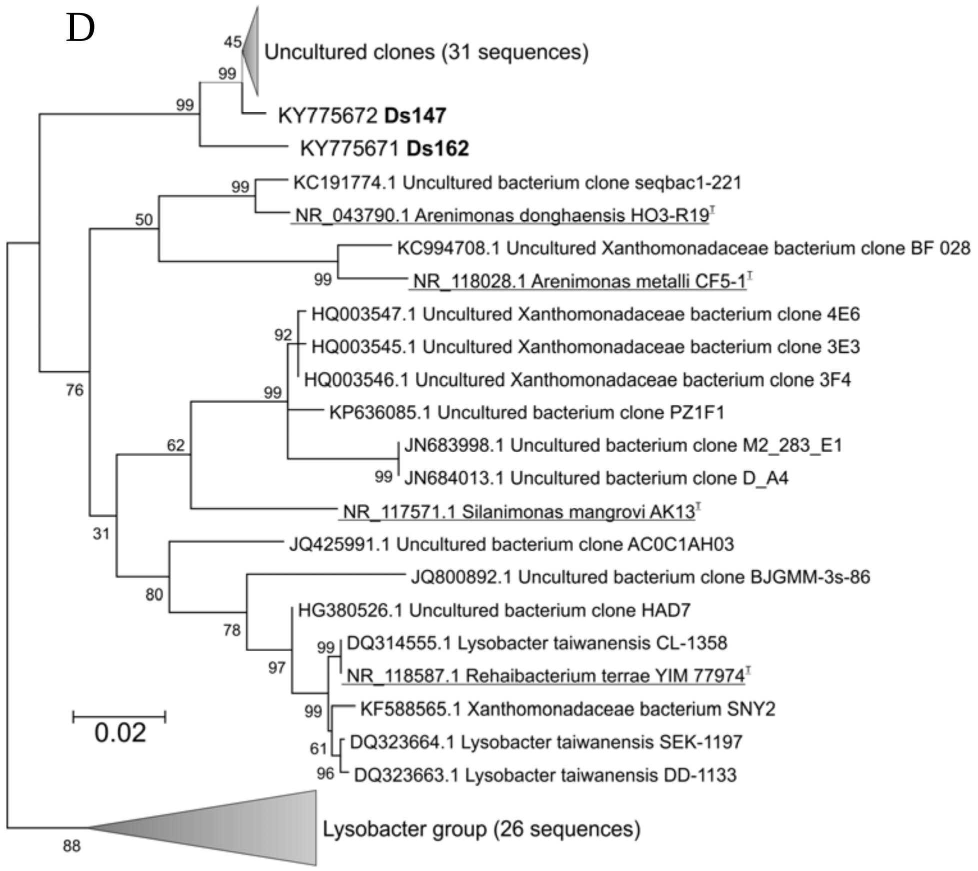
45
Uncultured clones (31 sequences)
99
99
KY775672 **Ds147**
KY775671 **Ds162**
99
KC191774.1 Uncultured bacterium clone seqbac1-221
50
NR_043790.1 Arenimonas donghaensis HO3-R19[T]
KC994708.1 Uncultured Xanthomonadaceae bacterium clone BF 028
99
NR_118028.1 Arenimonas metalli CF5-1[T]
HQ003547.1 Uncultured Xanthomonadaceae bacterium clone 4E6
92
HQ003545.1 Uncultured Xanthomonadaceae bacterium clone 3E3
HQ003546.1 Uncultured Xanthomonadaceae bacterium clone 3F4
76
99
KP636085.1 Uncultured bacterium clone PZ1F1
62
JN683998.1 Uncultured bacterium clone M2_283_E1
99
JN684013.1 Uncultured bacterium clone D_A4
NR_117571.1 Silanimonas mangrovi AK13[T]
31
JQ425991.1 Uncultured bacterium clone AC0C1AH03
JQ800892.1 Uncultured bacterium clone BJGMM-3s-86
80
HG380526.1 Uncultured bacterium clone HAD7
78
99
DQ314555.1 Lysobacter taiwanensis CL-1358
97
NR_118587.1 Rehaibacterium terrae YIM 77974[T]
99
KF588565.1 Xanthomonadaceae bacterium SNY2
0.02
61
DQ323664.1 Lysobacter taiwanensis SEK-1197
96
DQ323663.1 Lysobacter taiwanensis DD-1133
Lysobacter group (26 sequences)
88

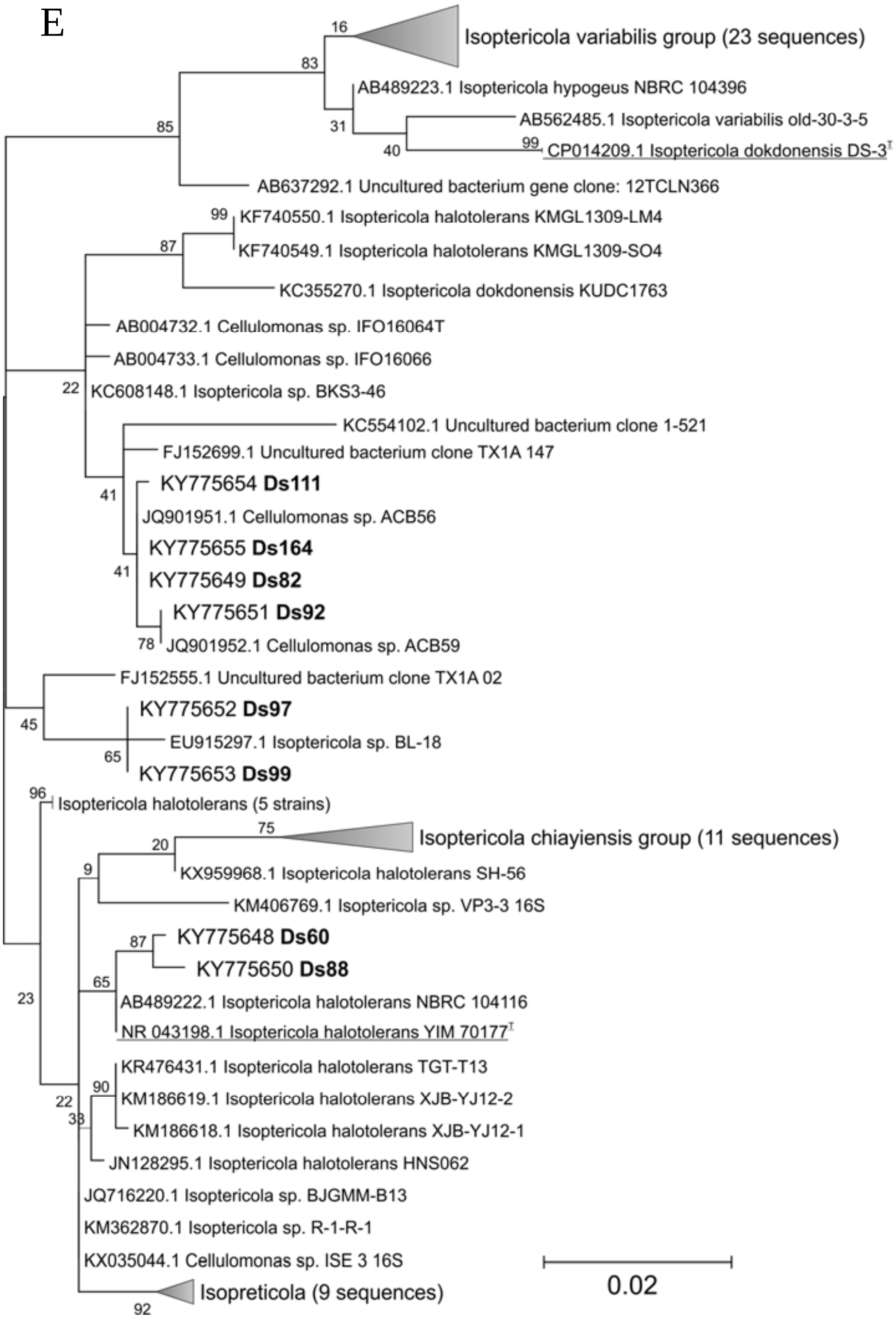

Suppl. Figure S1. Unrooted 16S rRNA gene sequence Maximum likelihood phylogenetic trees of the haloalkaliphilic isolates, possibly representing novel taxa, and its nearest relatives. All positions with less than 95 % site coverage were eliminated. There were a total of 1338 (A), 1394 (B), 1442 (C), 1373 (D) and 1103 (E) positions in the final datasets. The tree is drawn to scale, with branch lengths measured in the number of substitutions per site with corrections, associated with the model. Bootstrap values as percentages of 1000 repetitions are shown next to the branches. Type strains of validly published species are underlined. Genbank numbers are indicated at the beginning of each sequence designation.
